# A New Application of Lipid Nanoemulsions as Coating Agent, Providing Zero-Order Hydrophilic Drug Release from Tablets

**DOI:** 10.1155/2012/271319

**Published:** 2012-01-09

**Authors:** Nicolas Anton, Astrid de Crevoisier, Sabrina Schmitt, Thierry Vandamme

**Affiliations:** ^1^Faculty of Pharmacy, University of Strasbourg, 74 route du Rhin, BP 60024, 67401 Illkirch Cedex, France; ^2^CNRS UMR 7199, Laboratoire de Conception et Application de Molécules Bioactives, équipe de Pharmacie Biogalénique, 74 route du Rhin, BP 60024, 67401 Illkirch Cedex, France

## Abstract

The objective of the present investigation was to evaluate potential of nanoemulsions as a coating material for the tablets. The nanoemulsion of size less than 100 nm was prepared using a simple and low-energy spontaneous emulsification method. Conventional tablets containing theophylline as a model hydrophilic drug were prepared. The theophylline tablets were coated with the nanoemulsion using a fluid bed coater. The effect of different levels of the nanoemulsion coating on the theophylline release was evaluated. The theophylline tablets containing different levels of the nanoemulsion coating could be successfully prepared. Interestingly, the coating of tablet with the nanoemulsion resulted in zero-order release of theophylline from the tablets. The noncoated theophylline tablets release the entire drug in less than 2 minutes, whereas nanoemulsion coating delayed the release of theophylline from tablets. This investigation establishes the proof of concept for the potential of nanoemulsions as a coating material for tablets.

## 1. Introduction

The design and development of simple systems with the aim of delivery and controlled release of hydrophilic drugs administered through oral route are still a challenge. Compared to classical dosage forms, the goals for the development of such systems include maintaining of blood levels for the drug in a therapeutic window for a desired period. Such controlled drug-delivery systems present considerable advantage over conventional dosage forms, but they involve carrying out specific and complex technologies [1–12]. The most widespread systems giving modified releases are hydrophilic matrix carriers or hydrophilic coating matrix (e.g., on tablets). Pharmaceutically available polymers such as polymethacrylates (Eudragit RS100 and Eudragit S100), ethyl cellulose (EC), and hydroxypropyl methylcellulose (HPMC), as a single or mixed composition, are largely studied and used for this purpose [13–18]. The modified drug releases are actually a combination of several physical processes including, diffusion, polymer swelling, dissolution, or erosion [19–22].

The literature generally reports investigations on the impacts of the formulation parameters—for example, coatings levels, nature of solvent, nature of polymer and plasticizer, polymer particle size, polymer weight, degree of substitution and polymer concentration [5, 16–18, 23–26], and the processing parameters—air pressure and temperature on the physicochemical properties of the coated film, that is to say, on the drug release profiles. In this context, it has been shown that the drug release is mainly related to the physical behavior of the coating materials with regards to the release media (for instance, tensile strength, contact angle, and solubility) [5, 17, 27, 28]. It is easily understandable, since the drug release, in these coated systems, arises after the drug solvation and diffusion, and thus after the gradual swelling (i) firstly of the coating polymer and (ii) secondly of the vehicle (like a tablet). Accordingly, the solvated drug is released (e.g., by diffusion) through this swollen system towards the bulk phase. It is to be noted here that the swelling kinetics of the coating polymer is of prime importance and must be fast enough to prevent the tablet disintegration during this first phase of the process.

The particular case of zero order is of real interest, since it confers to the system, the ability to deliver a drug at a constant rate. Hence, a steady amount of drug is released over time, which, on the one hand, minimizes potential peak/trough fluctuations and side effects, and on the other hand, maximizes the time for which the drug concentrations remain within the therapeutic window. With the examples of hydrophilic matrix presented above, zero-order release profiles are the direct consequences of the Fickian diffusion of the drugs through a membrane (Fick's first law).

The zero-order release can also be induced by a specific swellable polymer coating technology. The numerous studies reported on these domains are focused on the formulation and processing parameters described above, for a single polymer or blend of various polymers. However, as a constant factor, these technologies still use polymers to create such a barrier between the drug and release media. This is precisely the novelty of our approach, since herein, we propose a new method, applicable to tablets to provide zero-order drug release profiles, by using lipids instead of polymers. This paper presents tablet lipid coating, based on a specific nanotechnology (lipid nanoemulsions), followed by a study of hydrophilic drug releases (theophylline), disclosure, and modeling the release mechanisms. The idea was to coat the tablets, by a lipid species, in order to create a lipid coating or lipid adsorbed layer, serving as barrier against the hydrophilic drug leakage. This was originally carried out by using a fluid-bed apparatus for spray-coating the tablets with an aqueous suspension of lipid nanodroplets, so-called nanoemulsions. Now, a question arises: why to use lipid nanosuspension for this purpose? The answer is simple, since (i) the lipid nanosuspension is able to penetrate the tablet microporous matrix, (ii) the huge homogeneity of these nanoemulsified dispersions will provide a very homogeneous coating, (iii) lipid nanoemulsions are very stable, easy to prepare and are fully compatible with the spray-coating technologies, and finally, (iv) the nanoemulsions formulated by low-energy methods (the case here) are very simple systems adaptable to industrial scaling-up and purposes.

Nanoemulsions are emulsions, in which the size of oil-in-water droplets are typically in nanorange, ranging between 20 and 300 nm [29–31]. The main advantage of nanoemulsions, as in our case, is their stability. Actually, due to their small size, the oil droplets behave typically as Brownian particles and do not interact with each others, resulting in their stability, for up to several months [32–34]. Accordingly, nanoemulsions are considered as particular tools for chemical and pharmaceutical applications, for example, allowing poorly soluble species in water to disperse in a stable way. Another application of nanoemulsion is their use as drug and/or contrast agent nanocarriers, potentially associated with surface functionalization for targeting applications.

In this context, the present study actually constitutes a novel and original application of nanoemulsions, along with a novel approach for the fabrication of oral modified drug-release systems. To summarize, this work presents a new technology for modifying the drug release of tablets. We describe the structures obtained and their links with the drug release kinetics, together with the physical processes involved.

## 2. Materials and Methods

### 2.1. Materials

Lactose monohydrate was provided by Danone (Paris, France) and microcrystalline cellulose (Emcocel 90 M) from JRS Pharma (Rosenberg, Germany). Corn starch, magnesium stearate, talc, and carmine red were obtained from Cooper (Melun, France). Colloidal silica (silica dioxide, Aerosil)was purchased from Evonik (Essen, Germany). Anhydrous theophylline was provided by Fagron (Saint-Denis, France). Food grade nonionic surfactants from BASF (Ludwigshafen, Germany), that is, Cremophor RH40 (polyoxyethylated-40 castor oil, hydrophilic-lipophilic balance, HLB ~14–16) were kindly provided by Laserson (Etampes, France) and used as received. Labrafil M1944CS used as oil phase in the formulation of nanoemulsions was obtained by Gattefossé (Saint-Priest, France). Finally, ultrapure water was obtained using the MilliQ filtration system, Millipore (Saint-Quentin-en-Yvelines, France).

### 2.2. Methods

#### 2.2.1. Tablets Fabrication

The formulation process and the composition of tablet followed classical pathways. In this study, two formulations named (A) and (B) were studied, differing in the proportions of binding (crosslinked microcrystalline cellulose) and disintegrating (corn starch) compounds. The quantities were as reported Table 1. 

Once mixed (lactose, cellulose, starch, carmine red, and theophylline), the powders were homogenized in a Turbula universal mixer (Basel, Switzerland) during 15 min. This was followed by the addition of magnesium stearate, and colloidal silica and the powder were further homogenized in the Turbula mixer for 30 seconds. Next, the powder is sieved through 1 mm meshes sieve and is then pressed with an alternative Frogerais press (Vitry-sur-Seine, France), using a 10 mm diameter hemispherical punch. The tablets thus formed are weighted, their hardness was measured and controlled with a durometer Erweka (Heusenstamm, Germany), and their friability evaluated with a specific apparatus PTF 10E, Pharma Test (Hainburg, Germany). For both formulation (A) and (B), the aimed tablet weight was fixed at 380 mg, and the aimed hardness was 90 and 190 N for the tablets (A) and (B), respectively.

#### 2.2.2. Nanoemulsion Formulation

Lipid nanoemulsions were formulated according to the low-energy emulsification process published elsewhere [33]. The nanoemulsion droplets were spontaneously formed by bringing into contact two phases: (i) the first was composed of lipid (liquid oil, Labrafil M1944CS) and a hydrophilic surfactant, both totally miscible in each other and gently homogenized at room temperature and (ii) the second phase was aqueous (pure water). Once these two liquid phases were mixed, the hydrophilic species were immediately solubilized by the aqueous phase, inducing the demixing of the oil following a spinodal decomposition, resulting in the nanoemulsion droplets. The nanoemulsion properties, that is, size and polydispersity, have been shown [33] to be closely related to the relative proportions between oil and surfactant. This parameter, so-called surfactant oil weight ratio (SOR = *w*_surfactant_/(*w*_surfactant_ + *w*_oil_) × 100) allows the droplet size and polydispersity index to be precisely controlled. In the present study, SOR was fixed at 40% as a representative formulation. Actually, in all the experiments presented here, the SOR (i.e., nanoemulsion droplets size) has no significant influence on the results as well as the release behavior. On the other hand, the relative proportion of water does not influence the nanoemulsion physicochemical properties or their size and PDI. This parameter is given by SOWR = *w*_surfactant_ + *w*_oil_/(*w*_surfactant_ + *w*_oil_ + *w*_water_) × 100, which was also fixed to 40%. The exact composition of the nanoemulsion used for coating of tablets is: oil: 24%; surfactant: 16%; water: 60%. The size distribution and polydispersity of nanoemulsions were assessed by dynamic light scattering (DLS) using a Malvern Nano ZS instrument (Malvern, Orsay, France). The Helium-Neon laser (4 mW) was operated at 633 nm with the scatter angle fixed at 173°, and the temperature was maintained at 25°C. The polydispersity index (PDI) is a measure of the broadness of the size distribution derived from the cumulants analysis of DLS. For a single Gaussian population with standard deviation *σ*, and mean size *x*_PCS_, then PDI = *σ* ^2^/*x*_PCS_^2^ is the relative variance of the distribution. The PDI discloses the quality of the dispersion, from values lower than 0.1 for acceptable measurements and good-quality colloidal suspensions, to values close to 1 for poor-quality samples, either with droplet sizes out of the colloidal range or with a very high polydispersity. Measurements were performed in triplicate, before and after the spray drying process (filtered at 0.45 *μ*m in the above case).

#### 2.2.3. Tablets Nanoemulsion Coating

The tablet coating was performed in a fluid bed “bottom spray” apparatus, Innojet Ventilus 2.5 (Steinen, Germany). 50 g of tablets are introduced in the chamber in which is also the rotating spray nose. The experiment was carried out according to the following experimental parameters: air flow:  76 m^3^/h; flux: 13%; temperature: 40°C. The weight increase due to the coating is regularly controlled, and the experiment is stopped when the desired nanoemulsion weight coating is obtained.

The upper coating level possible reached in these experiments was around 8%.

#### 2.2.4. Drug Release Profiles

Dissolution tests were performed in an automatized basket apparatus, Dissolutest Caleva BIO-DIS RRT 9 (Frankfurt, Germany). The basket volume is 250 mL, and the dissolution media was an aqueous solution of HCl 0.1 M, maintained at 37°C during 2 hours, as described in the European Pharmacopoeia (7th Ed.) for the delayed release dosage forms.

Aliquots are collected at regular time intervals fixed in function of the release kinetics. Then, the theophylline concentrations, and thus cumulative drug release, are measured at 288 nm by UV spectrophotometry, UV-2401 PC Schimadzu (Kyoto, Japan).

Before performing the measurements, the samples were filtered and diluted, which inhibits the absorption of the various excipients used. In that way, we prevented interference between the theophylline quantification and the absorption of the components of the nanoemulsions or of the tablets. Moreover, a blank test was also performed at 288 nm in absence of theophylline to validate of the measurements.

### 2.3. Scanning Electron Microscope (SEM)

The morphology of tablets (surface and interior) was evaluated by a scanning electron microscopy (Philips XL20, University of Strasbourg, plateforme de microscopie électronique, Institut de Génétique et de Biologie Moléculaire et Cellulaire). The specimens were mounted on the carbon support, coated with a palladium layer and analyzed at 20 kV.

## 3. Results

The first results concerns the tablet characterization, notably the controls described in the European Pharmacopoeia (7th Ed.).

These results are summarized in Table 2 and validate the dosage forms, compositions, and formulation processes.

The main difference between the two formulations arises in their hardness, and as expected, a higher amount of disintegrating compound reduces the hardness.

**Figure 1:**
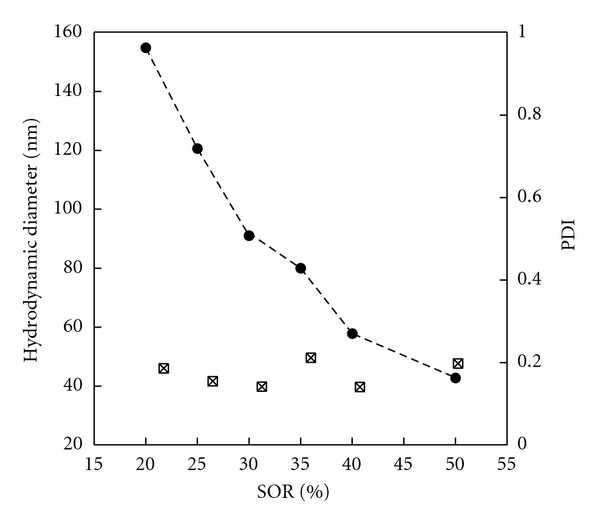
Nanoemulsions formulated with low-energy spontaneous emulsification. Surfactant = Cremophor RH40 oil = Labrafil M1944CS. Hydrodynamic diameter (filled circles) and polydispersity index (open squared) are plotted against the surfactant/oil weight ratio (SOR).

Another aspect of the earlier characterization lies in the study of the nanoemulsion formulation process. Hydrodynamic diameter and PDI were measured in function of the surfactant to oil ratio (SOR) defined above. The results are reported in Figure 1.

The global profile of the curves appears coherent with the ones expected for such self nanoemulsifying systems, with relatively monodisperse size distributions (PDI < 0.2). Accordingly, the representative formulation selected for the tablet coating was SOR = 40%, corresponding to *d*_*h*_ = 57.9 nm  and PDI = 0.14.

**Figure 2:**
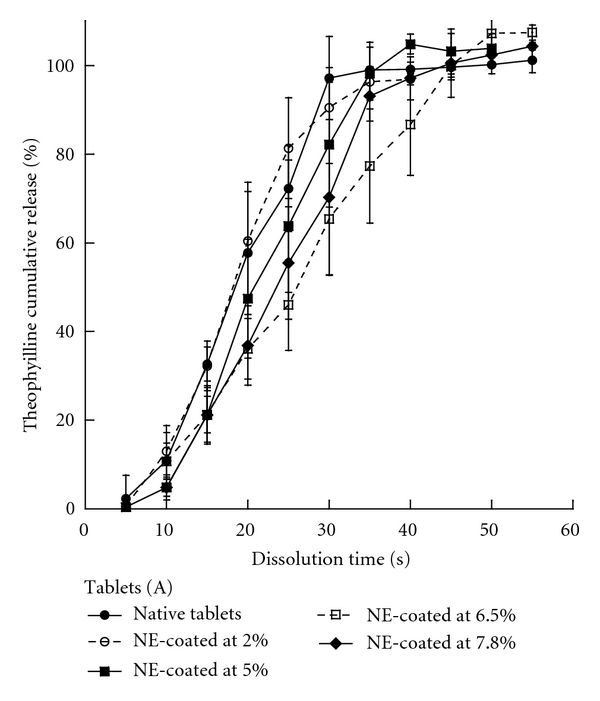
Theophylline release profiles from tablets (A) for different levels of nanoemulsion coating: 2%, 5%, 6.5% and 7.8%, and without coating (noncoated tablets).

**Figure 3:**
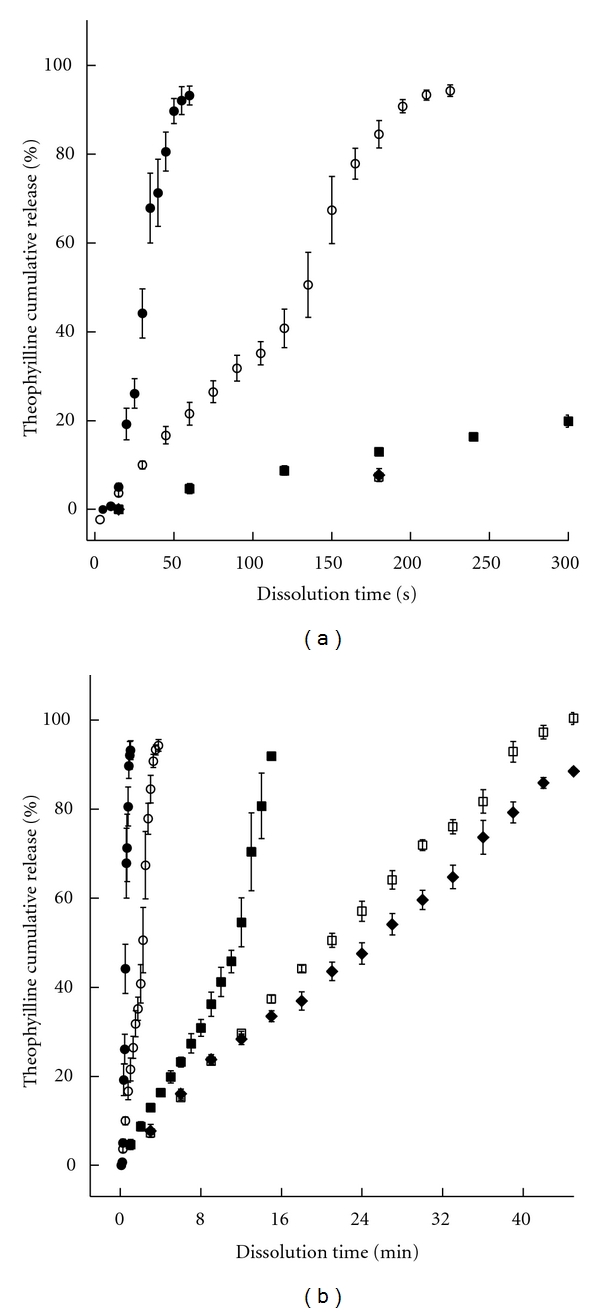
Theophylline release profiles from tablets (b) for different levels of nanoemulsion coating: 2%, 5.5%, 6%, and 7.6%, and without coating (noncoated tablets). The two graphs show the same results with different time scale, in order to emphasize the different release regimes arising for 2% and 5% (for which the frontiers between both are indicated by the arrows).

Once the tablets (A) and (B) coated with the nanoemulsion suspension, and at different given proportions, the followup of the theophylline release was performed. These results are reported in Figures 2 and 3, for the tablets (A) and (B), respectively.

It clearly appears that the theophylline release can be significantly modified by the intrinsic physical properties of the tablets associated with the lipid coating. In all the experiments, drug release from tablets (A) (Figure 2) was found to be independent of any coating, resulting in fast dissolutions within a minute. On the other hand, drug release from tablets (B) (Figure 3) were very sensitive to the amount of lipid coating. In addition, the curves for the coated tablets (B) show a linear release corresponding to the zero-order kinetics. This regimes, which is followed by a second nonlinear regime for 2.0% and 5.5%. The profiles are entirely linear up to the full release for higher coating amount, 6.0 and 7.6%, providing a zero order during 46 min and 1 h for these examples, respectively. For 2.0% and 5.5% the release profiles show that two regimes follow one another, one exhibits a zero-order release, while the other appears as a transitional drug release similar to the one in noncoated tablets (see details below). Arrows in the figure indicate the location of the frontier between both regimes.

**Figure 4:**
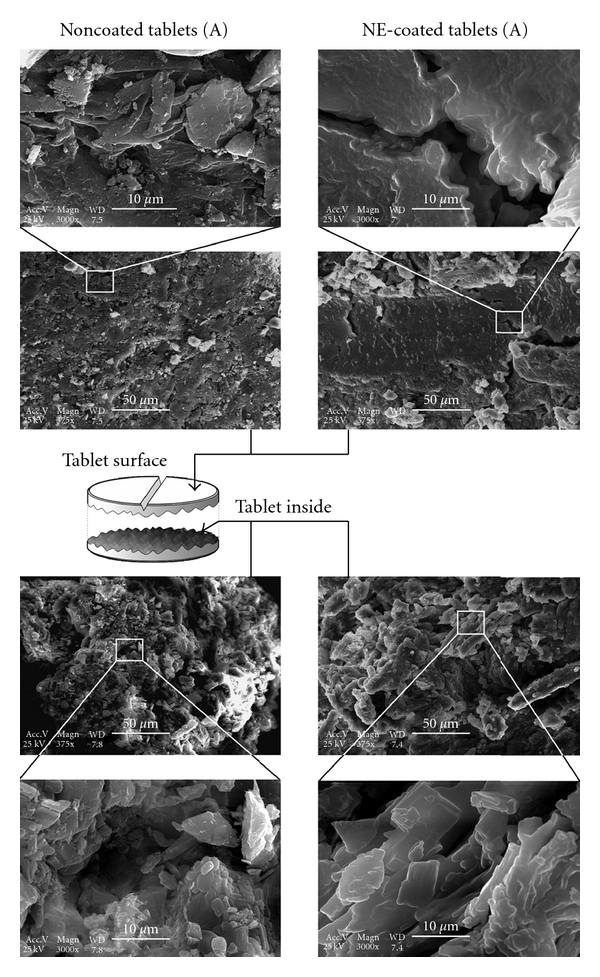
SEM micrographs of the tablets formulation (A). Observations performed on the tablet surface (top) and inside (bottom), for noncoated tablets and nanoemulsions coated (NE-coated).

**Figure 5:**
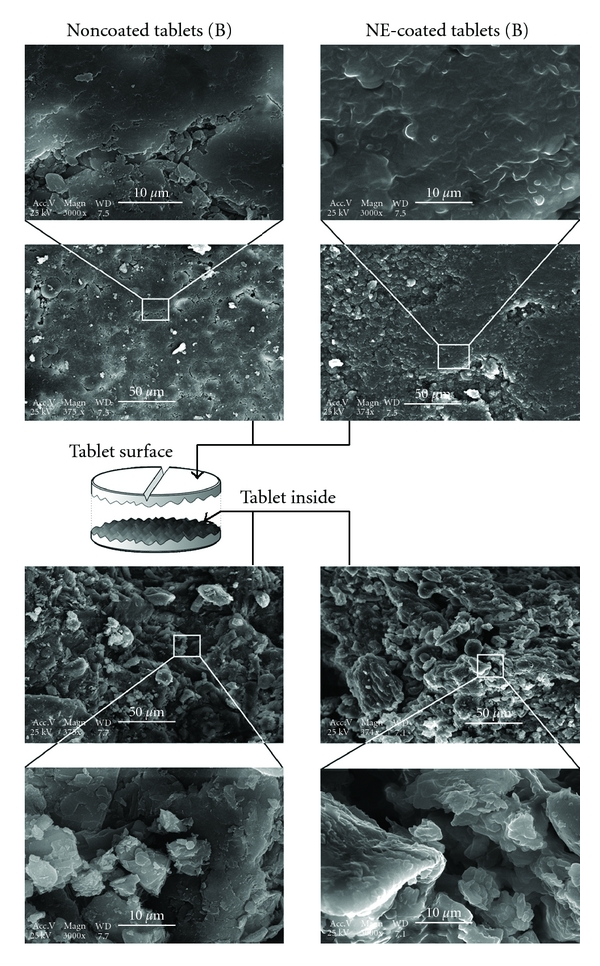
SEM micrographs of the tablets formulation (B). Observations performed on the tablet surface (top) and inside (bottom), for noncoated tablets and nanoemulsions coated (NE-coated).

In order to characterize the fine structure on the micrometric scale, the tablets were observed by scanning electron microscopy. The surface and interior of both coated and uncoated tablets, (A) and (B), were analyzed. The pictures are reported in the Figures 4 and 5, for the tablets (A) and (B), respectively.

In both cases (A) and (B), it clearly appears that the lipid coating creates a “smooth” layer on both the tablets surface and the tablets inside. The edges generated by the compression fully disappear after the coating. It means that the nanoemulsions are very homogeneously spread onto the available surface and also can penetrate the microporous tablet matrix during the spray-coating process, which can both be due to the nanometric scale of such a dispersed system. Another point lies in the difference between the formulations of (A) and (B), where the second one (B) was found to be more compact. This actually corroborates their difference in hardness (see Table 2) and contributes to explain the fundamental differences in the release profiles between both formulations (A) and (B).

## 4. Discussion

The main point of this study lies in the new and simple possibilities offered by lipid nanoemulsions (i) to integrate the microporous matrix of tablets (corroborated by the SEM pictures Figure 5), (ii) to homogeneously coat the surface, and (iii) to create a lipid barrier inducing a zero-order release mechanism in the formulation (B). One interpretation of this zero-order drug release could be the Fickian diffusion-based mechanism, considering that the lipid will create a “filter” or a membrane-like barrier against hydrophilic molecules. As a result, the theophylline molecules leakage from the tablet followed a linear release behavior as long as this lipid barrier is intact. This zero-order release process can be described as a constant regime, also called steady state diffusion. Considering the case of ideal thermodynamic system having a diffusion coefficient *D* which is independent from the concentration *C*, and having an unidimensional diffusion, this diffusion regime can be best described by the Fick's first law

(1) $$\begin{array}{c} J=\frac{dM_{t}}{Sdt}=-D\frac{dC}{dx}\;, \end{array}$$

where *J* is the flux, *S* the surface of the diffusion plane, and *x* is the distance of diffusion. Accordingly, this unidimensional equation can easily be adapted for the case of a spherical drug-delivery system of radius *R*_*e*_, composed of a diffusion-limiting barrier of thickness *R*_*e*_ − *R*_*i*_, giving the drug mass of the released *M*_t_ in function of time *t*, as reported in

(2) $$\begin{array}{c} M_{t}=\frac{R_{e}R_{i}\times 4\pi DKC_{0}}{R_{e}-R_{i}}\times t \end{array}\;,$$

where *K* is the partitioning coefficient between the lipid barrier and water, *C*_0_ is the difference in concentration between the both sides of the lipid barrier. When the amount of lipid is sufficient (e.g., Figure 3 cases 6 and 7.6%), this barrier appears to be strong enough to allow this linear behavior until the release of all the encapsulated drug amount. However, for intermediate concentrations (as observed in Figure 3 cases 2 and 5.5%), after a given time *t*_*α*_, this diffusion-limiting layer is dissolved or disaggregated, and a second phase of drug release occurs. This phase follows a “nonsteady state” diffusion regime for which the concentration gradient varies with time. This process is described in the general case by the Fick' second law, reported below:

(3) $$\begin{array}{c} \frac{dC}{dt}=D\frac{d^{2}C}{dx^{2}} \end{array}.$$

**Figure 6:**
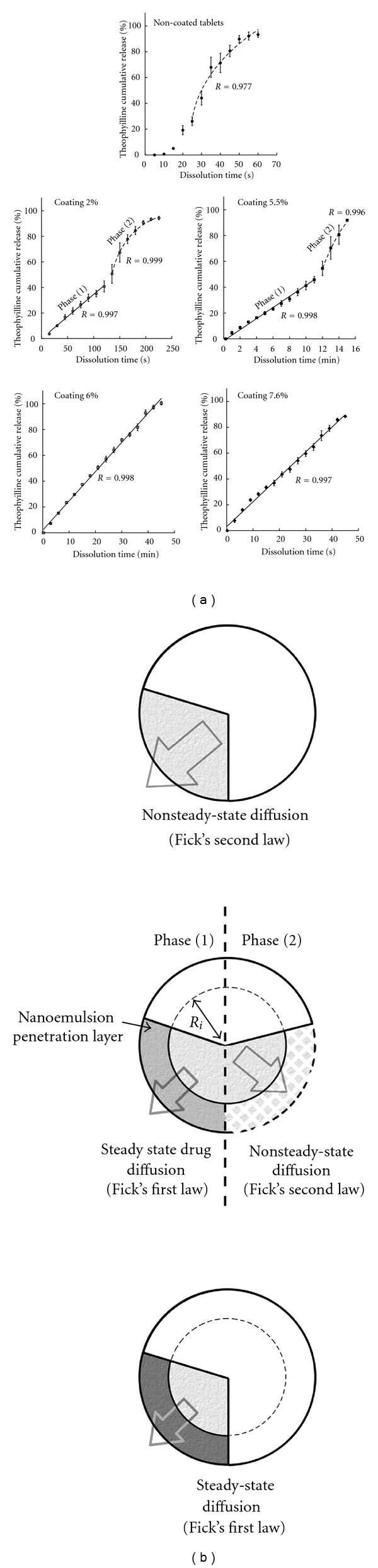
Interpretations of the drug release behaviors from Figure 3. Theophylline release from tablets (b), for different levels of nanoemulsion coating: 2%, 5.5%, 6%, and 7.6%, and noncoating tablets.

In the case of a spherical drug delivery matrix, this equation is adapted as shown below:

(4) $$\begin{array}{c} \frac{M_{t}}{M_{\infty }}=6{\left(\frac{D\left(t-t_{\alpha }\right)}{\pi R^{2}}\right)}^{1/2}-\frac{3D\left(t-t_{\alpha }\right)}{R^{2}}\;, \end{array}$$

where *M*_*∞*_ is the mass of the drug released at infinite time, *t*_*α*_ is the delay induced by the first zero-order release, and *R* is the sphere radius. This behavior is also found for the noncoated tablets, with a lag time *t*_*α*_ around 19 seconds due to the tablet hydration. It is interesting to note that the zero-order release profiles exhibit slopes (i.e., release speeds quantified below), decreasing with increasing amount of coating lipid. This detail confirms that the diffusion-based mechanism can be a correct interpretation of the zero-order phenomena compared to the other physical possible processes, for example, zero-order homogeneous erosion for which the release speed should be constant in similar experimental conditions. All the release profiles of the formulation (B) are fitted following these two models, and schematic illustrations of the mechanisms and tablets structures are reported in Figure 6.

The main results of a quantitative comparison of the different cases are reported in Table 3.

The theoretical models appear quite well in accordance with experimental results, which confirms the hypothesis ventured regarding the structures and the release processes. The higher the nanoemulsion coating level, the lower the release speed. If the coated lipid layer is considered globally constant, this behavior can be attributed to the decrease of the diffusion coefficient *D*, and thus to the decrease of the permeability *P* = *DK*/(*R*_*e*_ − *R*_*i*_). On the other hand, the time *t*_*α*_ in which this lipid layer is broken up also appears related to the coating amount. It follows therefrom that *t*_*α*_ indicates the transition between the two diffusion regimes (1) and (2) highlighted in Figure 6. The higher the coating amount, the more stable is the layer, being definitively stable for the examples of 6 and 7 wt.%. Finally, the last parameter *D*/*R* ^2^ characterizing the unsteady-state regimes, shows a gradual increase between the three first cases. As the natural trend for *D* is a decrease, the observed increase of *D*/*R* ^2^ emphasize a lowering of *R*, and thus of *R*_*i*_ with the lipid amount. To conclude, coating tablets with lipid nanoemulsions results in the fabrication of a surrounding lipid layer within the tablet, which is able to limit the drug diffusion, similar to a membrane. With the increase of the lipid coating wt.%, this layer become thicker and more stable. Compared now to the hydrophilic matrix discussed above, these systems, made from a fundamentally different technology, appear to present very similar properties.

As a last remark, let us focus on the formulation (A). Even if the coating process and tablet characterization are similar between (A) and (B), the drug release profiles do not have any similarities (Figure 2). Compared with the (B), the tablets (A) show much lower hardness (about half of that of B), which results in higher porosity. The impossibility to create an impermeable lipid layer results in identical drug release profiles whatever may be the coating amount. This can also be observed in the SEM pictures, of the tablet surfaces, which appear to be more compact and robust in the case of the formulation (B).

To finish, such a technology not only appears innovative under the fundamental point of view, since it is the first time that a zero-order release is obtained with a lipid coating, but also it appears interesting in term of industrial scaling up. On the one hand, the nanoemulsion generation method is extremely simple and can be performed only by mixing two liquids, and on the other hand, the method also appears cost effective since it avoids using very specific and expensive polymers for results which can be comparable.

## 5. Conclusion

This study presents for the first time the application of lipid nanosuspensions as coating agent for inducing a zero-order hydrophilic drug-release profile. To date, this result was only obtained by using hydrophilic polymeric matrix, and we showed here the proof of concept of this new technology. Lipid nanoemulsions generated by spontaneous nanoemulsifications were used as coating agent. The lipid nanodroplets were able to enter the lipid matrix, to coat the microporous network of the tablet, and to finally create a layer acting as barrier against the diffusion of hydrophilic drugs. This technology is simple, cost effective, and efficient, and we believe that it can open new perspectives for the fabrication of pharmaceutics and oral modified release-dosage forms.

## Figures and Tables

**Table 1:** Tablets composition (g).

	Tablets (A)	Tablets (B)
Lactose monohydrate	113.8	113.8
Microcrystalline cellulose	222.35	214.45
Corn starch	19.7	27.6
Magnesium stearate	5	5
Colloidal silica	5	5
Talc	2.5	2.5
Carmine red	0.05	0.05
Anhydrous theophylline	131.6	131.6

**Table 2:** Tablets characterization and Pharmacopoeia controls.

	Tablets (A)	Tablets (B)
Weight (mg)	383 ± 2	382 ± 3
Hardness (N)	84.2 ± 0.8	177.2 ± 0.5
Friability (%)	0.18 ± 0.04	0.12 ± 0.05
Desegregation (s)	19 ± 4	19 ± 2

**Table 3:** Experimental parameters obtained from the kinetics drug release of tablets (B) (see Figure 6). The release speeds reported (*dM*_*t*_/*dt*) correspond to the linear diffusion regime.

NE-coating	*v* = *dM*_*t*_/*dt* (10^−5^g · s^−1^)	*t*_*α*_	*D*/*R* ^2^(s^−1^)
non-coated	—	19 s	0.011
2 wt.%	38.9	122 s	0.236
5.5 wt.%	7.6	11 min	2.757
6 wt.%	4.2	—	—
7.6 wt.%	3.6	—	—
